# A perioperative infusion of sodium bicarbonate does not improve renal function in cardiac surgery patients: a prospective observational cohort study

**DOI:** 10.1186/cc11476

**Published:** 2012-08-16

**Authors:** Matthias Heringlake, Hermann Heinze, Maria Schubert, Yvonne Nowak, Janina Guder, Maria Kleinebrahm, Hauke Paarmann, Thorsten Hanke, Julika Schön

**Affiliations:** 1 Department of Anesthesiology, University of Lübeck, Ratzeburger Allee 160, Lübeck, D-23538, Germany; 2 Department of Cardiac and Thoracic Vascular Surgery, University of Lübeck, Ratzeburger Allee 160, Lübeck, D-23538, Germany

## Abstract

**Introduction:**

Cardiac-surgery-associated-acute-kidney-injury (CSA-AKI) is associated with increased morbidity and mortality. Recent data from patients undergoing on-pump coronary artery bypass grafting suggest that a perioperative infusion of sodium-bicarbonate may decrease the incidence of CSA-AKI. The present study aims to analyze the renoprotective effects of a 24h infusion of sodium-bicarbonate in a large, heterogeneous group of cardiac surgical patients

**Methods:**

Starting in 4/2009, all patients undergoing cardiac surgery at our institution were enrolled in a prospective trial analyzing the relationship between preoperative cerebral oxygen saturation and postoperative organ dysfunction. We used this prospectively sampled data set to perform a cohort analysis of the renoprotective efficiency of a 24h continuous perioperative infusion of sodium-bicarbonate on the incidence of CSA-AKI that was routinely introduced in 7/2009. After exclusion of patients with endstage chronic kidney disease, off-pump procedures, and emergency cases, perioperative changes in renal function were assessed in 280 patients treated with a perioperative infusion of 4 mmol sodium-bicarbonate / kg body weight in comparison with a control cohort of 304 patients enrolled from April to June in this prospective cohort study.

Postoperative changes in urine flow, plasma creatinine, estimated creatinine clearance, and the need for renal replacement therapy were determined according to AKI injury network criteria. Concomitantly, hemodynamics, treatments, complications, and clinical outcomes were recorded. Univariate statistical analyses were performed para- and nonparametrically, as appropriate.

**Results:**

With the exception of a lower prevalence of a history of myocardial infarction and a lower preoperative use of intravenous heparin in the bicarbonate-group, no significant between group differences in patient demographics, surgical risk, type, and duration of surgery were observed. Patients in the bicarbonate group had a lower mean arterial blood pressure after induction of anesthesia, needed more fluids, more vasopressors, and a longer treatment time in the high dependency unit. Despite a higher postoperative diuresis, no differences in the incidence of AKI grade 1 to 3 and the need for renal replacement were observed.

**Conclusions:**

Routine perioperative administration of sodium bicarbonate failed to improve postoperative renal function in a large population of cardiac surgical patients.

## Introduction

Acute kidney injury (AKI) is a frequent complication in patients undergoing cardiac surgery [1,2]. With respect to the multifactorial nature of this complication and the consequences for short- and long-term prognosis [3] there is an increasing clinical and scientific interest in this topic that has now been specifically referred to as cardiac surgery-associated kidney injury (CSA-AKI) [4].

Recent work suggests that the renal excretion of hemo- and myoglobin, as a consequence of the destruction of blood cells during cardiopulmonary bypass and tissue injury during prolonged periods of hypoperfusion, and the subsequent development of a pigment nephropathy, may play a pathophysiologically important role in CSA-AKI [5]. In support of this concept and with respect to the fact that urine alkalization with sodium bicarbonate (BIC) has traditionally been used to ameliorate the consequences of hemo- and myoglobinuria [6], Haase and coworkers have recently shown that perioperative 24-h treatment with BIC reduced the incidence of AKI in a double-blind pilot trial including 100 patients undergoing on-pump cardiac surgery [7]; findings that are currently being prospectively validated in 500 patients (the BIC-NC study; Clinical trials identifier NCT00672334)

With respect to the enormous impact of postoperative renal dysfunction on outcome in cardiac surgical patients, the sound pathophysiological basis, and positive historical experiences with urine alkalization in our center we decided not to await the results of the ongoing multicentre trial and implemented the concept of perioperative BIC infusion in July 2009 at our institution. With respect to the fact that since April 2009 almost all patients undergoing cardiac surgery at the University of Lübeck were enrolled in a prospective observational trial analyzing the association between preoperative cerebral oxygen saturation and postoperative organ dysfunction, we chose to use this database also to analyze the effects of routine BIC application on the perioperative changes in renal function and the incidence of AKI in this prospective, observational cohort study.

## Materials and methods

Following approval by the local ethical committee (Ethikkommission der Universität zu Lübeck, Lübeck, Germany), all patients scheduled for cardiac surgery at the University of Lübeck from 1 April 2009 to 31 December 2009 were screened for participation in a prospective, observational trial on the relationship between preoperative cerebral oxygenation and postoperative organ dysfunction. The only exclusion criterion was age less below 18 years. Written informed consent was obtained from all elective and urgent patients as well as emergency patients who were able to communicate. In the case of sedated and/or intubated patients scheduled for emergency surgery, consent was obtained from the next of kin and reconfirmed after recovery.

From July 2009, all patients were treated with a perioperative infusion of BIC, and the treatment was maintained until the first results of the present study were available in December 2010. Following approval by the local ethical committee of amendment 5 to the primary request (reference number, 07-146), we specifically analyzed the perioperative changes in renal function and general patient outcomes according to the use or non-use of BIC. After exclusion of patients with end-stage chronic kidney disease, off-pump procedures, and emergency cases, perioperative changes in renal function were assessed in 280 patients treated with BIC in comparison with a control cohort (CON) of 304 patients enrolled from April to June. In addition to the standard treatment, plasma samples for determination of N-terminal pro B-type natriuretic peptide (NTproBNP) and high-sensitivity troponin-T (hsTNT) were drawn immediately before surgery.

The perioperative infusion of BIC was adapted from Haase and coworkers [(7)]. A bolus of 0.5 mmol/Kg body weight (BW) in a balanced electrolyte infusion (500 ml) was infused within 30 minutes immediately after induction of anesthesia. Thereafter, a maintenance infusion of 0.15 mmol/Kg BW/h in 500 ml dextrose in water (5%) was applied within 24 h, giving a total dose of 4 mmol BIC/Kg BW during 24 h. Anesthesiological, surgical, and intensive care treatment followed the standardized algorithms established at the Department of Anesthesiology and the Department of Thoracic Vascular and Cardiac Surgery of the University of Lübeck.

### Anesthesiological and intensive care treatments

Following oral premedication with 3.75 to 7.5 mg midazolam, and transfer to the operating room, sensors for the determination of cerebral oxygen saturation (ScO2) with an INVOS^®^ 5100 monitor (Somanetics, Troy, MI, USA) were applied bi-hemisperically before induction of anesthesia. Baseline ScO2 was determined in the resting state when breathing room air. General anesthesia was induced with sufentanil 0.5 μg/Kg BW, etomidate 0.2 to 0.4 mg/Kg BW and maintained at 0.8 to 1.0 minimal alveolar concentration of sevoflurane and remifentanyl at 0.2 to 0.4 μg/Kg BW/min, with the goal of early postoperative extubation. Muscle relaxation was achieved with rocuronium bromide 0.6 mg/Kg BW. During cardiopulmonary bypass (CPB), propofol at a dose of 4 to 5 mg/Kg BW/h was applied, since at that time volatile anesthetics could not be given via the CPB circuit. After surgery, all patients were transferred to the ICU, and after normothermia had been achieved, were weaned from the respirator as soon as possible.

Hemodynamic therapy aimed at a mean arterial blood pressure (MAP) between 60 and 90 mmHg, a heart rate (HR) between 60 and 90 bpm, a central venous pressure (CVP) between 10 and 15 mmHg, and central venous oxygen saturation (ScvO2) greater than 70%. In patients monitored with a pulmonary artery catheter, hemodynamic therapy was titrated to achieve a cardiac index > 2.2 l/min/m2 and mixed venous oxygen saturation (SvO2) greater than 65% [8]. Pulmonary arterial pressure (PAP) was used to guide hemodynamic therapy for right heart and pulmonary vascular function.

Fluid therapy was performed by balanced cristalloid (Sterofundin ISO 1/1; BBraun; Melsungen, Germany) and colloidal (Volulyte^®^, Fresenius, Germany; Gelafundin^®^, BBraun, Melsungen, Germany) fluids, as suggested by a recent guideline [8].

### Surgical and cardiopulmonary bypass treatments

All surgeries were performed with CPB in moderate or deep hypothermia (according to the preferences of the surgeon and the scheduled type of surgery). Cardioplegic arrest was achieved by blood cardioplegia and repeated every 20 minutes. In coronary-artery bypass grafting (CABG), a single crossclamp was generally used. Non-pulsatile perfusion was performed during CPB. Pump flow, oxygen flow, and MAP were adjusted to maintain ScO2 levels higher than 50% absolute.

### Clinical treatments and outcomes

Patients were discharged from the ICU to the intermediate care unit (IMC) if they were hemodynamically stable with only moderate inotropic and/or vasopressor support and were breathing spontaneously without the need for non-invasive ventilation. They were discharged to the surgical ward if they had been completely weaned from vasoactive or inotropic drugs, showed no signs of a relevant organ dysfunction (that is, pneumonia, AKI, severe neurological dysfunction, etcetera), and were free of delirium.

A perioperative myocardial infarction was defined as a relevant increase in myocardial necrosis markers (creatinine-kinase and creatinine-kinase MB fraction) in combination with the development of typical electrocardiographic findings or the detection of a new wall motion abnormality by echocardiography.

### Indication and performance of renal replacement therapy

Indications for renal replacement therapy were as follows: potassium equal to or higher than 6 mmol/L despite forced diuresis or prolonged treatment with glucose-insulin solution; fluid overload with imminent or present extrarenal organ dysfunction not responsive to moderate doses of diuretics (that is, more than 80 mg/d torasemide), severe low cardiac output state with persistent oliguria despite, or inadequate reaction to a bolus of a loop diuretic (furosemide 40 mg, torasemide 20 mg).

Renal replacement therapy was performed as veno-venous hemodiafiltration in postdilution mode with a blood flow of 150 to 200 ml/min, an ultrafiltration dose of 20 ml/Kg BW/h, and a dialysis dose of 15ml/Kg/h.

### Statistical analysis

Data entry and analysis were performed with MedCalc 12.1.4. for Windows. Following Kolmogorov-Smirnov testing for normality of distribution, data were analyzed by parametric and non-parametric tests, as appropriate. Accordingly, if not stated otherwise, data are presented as mean and 95% confidence interval for the mean, or median and 95% confidence interval for the median. A *P*-value < 0.05 was considered to indicate statistical significance.

The severity of postoperative kidney dysfunction was quantified according to the criteria of AKI as suggested by the Acute Kidney Injury Network [9]. Additionally, and with respect to the ongoing prospective trial on the effects of BIC on renal function in cardiac surgery patients (BIC-NC study; Clinical trials identifier NCT00672334), we determined the number of patients showing a postoperative increase in plasma creatinine between 25 and 50%.

## Results

### Comparability of the study cohorts

With the exception of a lower prevalence of a history of myocardial infarction (18.6% vs. 26.3%) and a lower preoperative use of intravenous heparin in the BIC group, the study cohorts were highly comparable regarding baseline demographics, surgical procedures, and surgical core data (Table 1, 2, and 3). No significant between-group differences were observed in the additive Euroscore, plasma levels of NTproBNP, high-sensitivity troponin-T, creatinine, and estimated glomerular filtration rate (eGFR) as accepted risk factors for postoperative renal dysfunction.

**Table 1:** Demographic data and preoperative risk stratification

	Total cohort	Control - no bicarbonate	Intervention - bicarbonate	Significance, *P*-value
**Number**	584	304 (52.1%)	280 (47.9%)	
**Demographics**				
Age, years	68.5 (67, 69)	68.5 (67, 70)	68.5 (67, 70)	0.675
Male	381 (65.2%)	201 (66.1%)	180 (64.3%)	0.706
Female	203 (34.8%)	103 (33.9%)	100 (35.7%)	
Height, cm	171 (170, 172)	171 (170, 173)	171 (170, 172)	0.741
Weight, Kg	81.3 (79.9, 82.6)	82.3 (80.6, 84.1)	80.1 (78.1, 82.1)	0.103
BMI (Kg/m^2^)	27.2 (26.8, 27.7)	27.4 (26.8, 27.9)	26.9 (26.5, 27.8)	0.098
**Cardiovascular risk factors**				
Diabetes mellitus	158 (27.1%)	72 (23.8%)	86 (30.7%)	0.073
Arterial hypertension	484 (82.9%)	250 (82.2%)	234 (83.6%)	0.751
Smoking (former or present smoker)	246 (42.3%)	125 (41.5%)	121 (43.2%)	0.744
Hyperlipidemia	410 (70.2%)	217 (71.4%)	193 (68.9%)	0.578
Arterial vascular disease	135 (23.1%)	62 (20.4%)	73 (26.1%)	0.127
PAH (systolic > 60 mmHg) (n = 223)	32 (14.3%)	12 (11.1%)	20 (17.4%)	0.252
PAH (systolic > 40 mmHg) (n = 223)	112 (50.2%)	56 (51.9%)	56 (48.7%)	0.736
**NYHA I/II**	348 (59.6%)	184 (60.5%)	164 (58.6%)	0.692
**NYHA III/IV**	236 (40.4%)	120 (39.5%)	116 (41.4%)	
LVEF < 30%	32 (5.5%)	18 (6.0%)	14 (5.0%)	0.658
LVEF 30 to 50%	139 (24.0%)	68 (22.6%)	71 (25.5%)	
LVEF > 50%	408 (70.5%)	215 (71.4%)	193 (69.4%)	
ASA	3 (3, 3)	3 (3, 3)	3 (3, 3)	0.801
Add. EuroSCORE	5 (5, 5)	5 (5, 5)	4 (5, 5)	0.455
Add. EuroSCORE 0 to 2	134 (22.9%)	63 (20.7%)	71 (25.4%)	0.300
Add. EuroSCORE 3 to 5	205 (35.1%)	114 (37.5%)	91 (32.5%)	
Add. EuroSCORE > 5	245 (42.0%)	127 (41.8%)	118 (42.1%)	
**Other risk factors**				
Pulmonary disease	91(15.6%)	46 (15.1%)	45 (16.1%)	0.843
Neurological disease	70 (12.0%)	36 (11.8%)	34 (12.1%)	0.988
Left main stem disease	84 (14.4%)	42 (13.8%)	42 (15.0%)	0.772
Previous cardiac or thoracic vascular surgery	63 (10.8%)	31 (10.2%)	32 (11.4%)	0.730
History of myocardial infarction	132 (22.6%)	80 (26.3%)	52 (18.6%)	0.033
Troponin-T positive status	14 (2.4%)	11 (3.6%)	3 (1.1%)	0.082
History of cardiogenic shock or acute decompensated heart failure	71 (12.2%)	32 (10.6%)	39 (13.9%)	0.278
**Preoperative physiological profile**				
MAP, mmHg	97 (96, 98)	98 (96, 100)	96 (93, 99)	0.305
Heart rate, bpm	68 (67, 70)	68.5 (67, 70)	68 (67, 70)	0.918
Mean ScO2_room_, %	63 (62, 63)	62 (62, 64)	63 (62, 64)	0.695
Mean ScO2_ox_, %	68 (67, 68.17)	68 (67, 69)	68 (67, 69)	0.926
ScO2_minox_, %	66 (65, 67)	66 (65, 67)	65 (65, 67)	0.924
NTproBNP, pg/ml (n = 389)	455 (355, 519)	392 (312, 496)	517 (391, 686)	0.234
hsTNT, pg/ml (n = 389)	9 (8, 11)	9 (8, 13)	9 (7, 11)	0.850

Demographics, risk factors, and preoperative physiological status are shown for patients undergoing elective, on-pump cardiac surgery with or without a perioperative infusion of bicarbonate. BMI: body mass index; PAH: pulmonary arterial hypertension; NYHA: New York Heart Association classification; LVEF: left ventricular ejection fraction; ASA: American Society of Anesthesiology classification; Add.: additive; MAP: mean arterial pressure. ScO2: cerebral oxygen saturation determined bi-hemispherically by near-infrared spectroscopy; ScO2_room_: mean of both hemispheres determined when breathing room air; ScO2_ox_: mean of both hemispheres determined during application of oxygen-enriched air. ScO2_minox_: minimal ScO2 from the left or right hemisphere determined during application of oxygen-enriched air; NTproBNP: N-terminal pro B-type natriuretic peptide; hsTNT: high-sensitivity troponin-T. Data are given as absolute numbers (percentage) for nominal variables and median (95% confidence interval).

**Table 2:** Surgical procedures and performance

	Total cohort	Control - no bicarbonate	Intervention - bicarbonate	Significance, *P*-value
**Number**	584	304 (52.1%)	280 (47.9%)	
**Type of procedure**				
CABG	222 (38.1)	110 (36.3%)	112 (40.0%)	0.160
Valve surgery +/- varia	176 (30.1%)	93 (30.7%)	82 (28.9%)	
CABG +/- valve +/- varia	120 (20.6%)	71 (23.4%)	49 (17.5%)	
Aortic surgery +/- valve +/- varia	54 (9.3%)	21 (6.9%)	33 (11.8%)	
Various procedures	10 (1.7%)	8 (2.6%)	3 (1.1%)	
Aortic surgery +/- CABG	2 (0.3%)	1 (0.3%)	1 (0.4%)	
**Duration of surgery, minutes**	254 (247, 261)	249 (242, 260)	258 (250, 268)	0.406
**Duration of CPB, minutes**	115 (110, 119)	115 9 (107, 122)	115 (109, 120)	0.756
**Crossclamp time, minutes**	89.5 (84, 93)	88.5 (82, 93)	90 (83, 96)	0.539
**ECLS**	7 (1.2%)	3 (1.0%)	4 (1.4%)	0.913
**IABP**				
No IABP	556 (95.2%)	289 (95.1%)	267 (95.4%)	0.445
IABP before CPB	6 (1.0%)	2 (0.7%)	4 (1.4%)	
IABP during CPB	8 (1.4%)	6 (2.0%)	2 (0.7%)	
IABP after CPB	8 (1.4%)	3 (1.0%)	5 (1.8%)	
IABP on ICU	6 (1.0%)	4 (1.3%)	2 (0.7%)	
**Circulatory arrest**	21 (3.6%)	13 (4.3%)	8 (2.9%)	0.485
**Lowest body temperature, °C**	32 (32, 32)	32 (32, 32)	32 (32, 32)	0.615

Surgical procedures and surgical core data are shown for patients undergoing elective on-pump cardiac surgery with or without a perioperative infusion of bicarbonate. CABG: coronary artery bypass grafting; CPB: cardiopulmonary bypass; ECLS: extracorporeal life support; IABP: intra-aortic balloon pump. Data are given as absolute numbers (percentage) for nominal variables and as median (95% confidence interval).

**Table 3:** Preoperative, intraoperative and postoperative therapies

	Total cohort	Control - no bicarbonate	Intervention - bicarbonate	Significance, *P*-value
**Number**	584	304 (52.1%)	280 (47.9%)	
**Preoperative treatments**				
Beta-blocking agents	442 (75.8%)	234 (77.0%)	208 (74.6%)	0.558
ACE-inhibitors/ARB	424 (72.7%)	225 (74.0%)	199 (71.3%)	0.526
Diuretics	328 (56.4%)	173 (56.9%)	155 (55.8%)	0.844
Vasodilators	209 (35.8%)	110 (36.2%)	99 (35.5%)	0.929
Phenprocoumon	11 (1.9%)	4 (1.3%)	7 (2.5%)	0.451
Aspirine	373 (64.1%)	203 (66.8%)	170 (61.2%)	0.185
Clopidogrel	53 (9.1%)	24 (7.9%)	29 (10.4%)	0.366
Heparin, intravenous	95 (16.3%)	62 (20.4%)	33 (11.8%)	0.007
**Lipid-lowering drugs**	356 (61.1%)	197 (64.8%)	159 (57.0%)	0.065
Insulin	64 (11.0%)	30 (9.9%)	34 (12.2%)	0.446
Antibiotics	64 (11.0%)	33 (10.9%)	31 (11.1%)	0.973
Nitrates, intravenous	29 (5.0%)	17 (5.6%)	12 (4.3%)	0.599
Levosimendan	16 (2.7%)	7 (2.3%)	9 (3.2%)	0.669
**Intraoperative fluids**				
Cristalline fluids, ml	1,948 (1,903, 1,993)	1,982 (,1921, 2,042)	1,911 (1,844, 1,978)	0.120
Colloidal fluids, ml	800 (756, 843)	791 (730, 852)	809 (748, 870)	0.680
**Postoperative fluids within 24 h**				
Cristalline fluids, ml	2,719 (2,667, 2,771)	2,590 (2,519, 2661)	2,859 (2,786, 2,933)	< 0.0001
Colloidal fluids, ml	1,476 (1,412, 1,541)	1,404 (1,314, 1,494)	1,555(1,462, 1,647)	0.022
**Intraoperative blood transfusion**				
PRC, patients transfused	314 (53.8%)	166 (54.6%)	148 (52.9%)	0.734
units transfused	1 (0, 1)	1 (0, 1)	1 (0, 1)	0.925
FFP, patients transfused	38 (6.5%)	20 (6.6%)	18 (6.4%)	0.925
units transfused	0 [0-0]	0 [0-0]	0 [0-0]	0.871
TC, patients transfused	30 (5.1%)	16 (5.3%)	14 (5.0%)	0.965
units transfused	0 (0, 0)	0 (0, 0)	0 (0, 0)	0.980
Cellsaver, number patients	22 (3.8%)	15 (4.9%)	7 (2.5%)	0.185
**Postoperative blood transfusion**				
PRC, patients transfused	253 (43.3%)	129 (42.4%)	124 (44.3%)	0.713
units transfused	0.00 (0.00, 0.00)	0.00 (0.00, 0.00)	0.00 (0.00, 0.88)	0.505
FFP, patients transfused	43 (7.4%)	22 (7.2%)	21 (7.5%)	0.971
units transfused	0.00 (0.00, 0.00)	0.00 (0.00, 0.00)	0.00 (0.00, 0.00)	0.907
TC, patients transfused	43 (7.4%)	22 (7.2%)	21 (7.5%)	0.980
units transfused	0.00 (0.00, 0.00)	0.00 (0.00, 0.00)	0.00 (0.00, 0.00)	0.881
**Intraoperative vasoactive and inotropic treatments**				
Levosimendan, n (%)	24 (4.1%)	11 (3.6%)	13 (4.6%)	0.679
Before CPB	23 (3.9%)	11 (3.6%)	12 (4.3%)	0.531
After CPB	1 (0.2%)	0 (0.0%)	1 (0.4%)	
Noradrenaline, n (%)	522 (89.4%)	268 (88.2%)	254 (90.7%)	0.386
< 0.3 mg/h	261 (44.7%)	148 (48.7%)	113 (40.4%)	0.069
0.3 to 0.6 mg/h	187 (32.0%)	87 (28.6%)	100 (35.7%)	
> 0.6 mg/h	74 (12.7%)	33 (10.9%)	41 (14.6%)	
Vasopressin, n (%)	49 (8.4%)	19 (6.2%)	30 (10.7%)	0.073
< 3 U/h	33 (5.7%)	11 (3.6%)	22 (7.9%)	0.109
3 to 6 U/h	15 (2.6%)	8 (2.6%)	7 (2.5%)	
> 6 U/h	1 (0.2%)	0 (0.0%)	1 (0.4%)	
Dobutamin, n (%)	330 (56.5%)	178 (58.6%)	152 (54.3%)	0.339
< 15 mg/h	130 (22.3%)	70 (23.0%)	60 (21.4%)	0.278
15 to 30 mg/h	170 (29.1%)	96 (31.6%)	74 (26.4%)	
> 30 mg/h	30 (5.1%)	12 (3.9%)	18 (6.4%)	
**PDE-III inhibitors, n (%)**	249 (42.6%)	121 (39.8%)	128 (45.7%)	0.174
Low dose	35 (6.0%)	14 (4.6%)	21 (7.5%)	0.126
Moderate dose	198 (33.9%)	96 (31.6%)	102 (36.4%)	
High dose	16 (2.7%)	11 (3.6%)	5 (1.8%)	
**Postoperative vasoactive and inotropic treatment, n (%)**				
Levosimendan	24 (4.1%)	12 (3.9%)	12 (4.3%)	0.998
Noradrenalin	494 (84.6%)	246 (80.9%)	248 (88.6%)	0.015
< 0.3 mg/h	262 (44.9%)	147 (48.4%)	115 (41.1%)	0.0013
0.3 to 0.6 mg/h	140 (24.0%)	58 (19.1%)	82 (29.3%)	
> 0.6 mg/h	92 (15.8%)	41 (13.5%)	51 (18.2%)	
Vasopressin	64 (11.0%)	29 (9.5%)	35 (12.5%)	0.312
< 3 U/h	49 (8.4%)	19 (6.2%)	30 (10.7%)	0.116
3 to 6 Uh	13 (2.2%)	8 (2.6%)	5 (1.8%)	
> 6 U/h	2 (0.3%)	2 (0.7%)	0 (0.0%)	
Dobutamin	386 (66.1%)	207 (68.1%)	179 (63.9%)	0.330
< 15 mg/h	184(31.5%)	97 (31.9%)	87 (31.1%)	0.728
15 to 30 mg/h	164 (28.1%)	90 (29.6%)	74 (26.4%)	
> 30 mg/h	38 (6.5%)	20 (6.6%)	18 (6.4%)	
PDE III inhibitors	312 (53.5%)	156 (51.3%)	156 (55.9%)	0.304
Low dose	60 (10.3%)	32 (10.5%)	28 (10.0%)	0.665
Moderate dose	224 (38.4%)	110 (36.2%)	114 (40.9%)	
High dose	28 (4.8%)	14 (4.6%)	14 (5.0%)	

Perioperative pharmacological treatments are shown for patients undergoing elective on-pump cardiac surgery with or without a perioperative infusion of bicarbonate. ACE-inhibitors: angiotensin converting enzyme inhibitors; ARB: angiotensin receptor blocker; PRC: packed red cells; FFP: fresh frozen plasma; TC: thrombocyte concentrate; PDE-III inhibitors; phosphodiesterase III inhibitors. Data are given as absolute numbers (percentage) for nominal variables, and as median (95% confidence interval) for non-normally distributed data, and mean (95% confidence interval) for normally distributed variables.

### Hemodynamics and metabolism

Despite a comparable preoperative baseline, MAP after induction of anesthesia was significantly lower in the BIC group. Comparably, mean PAP was lower in these patients upon arrival on the ICU. No further significant between-group differences in hemodynamics were observed (Table 4). Maximal postoperative blood glucose and lactate levels, as well as maximal Ph, were higher in the BIC group (Table 5).

**Table 4:** Intraoperative and postoperative hemodynamics

	Total cohort	Control - no bicarbonate	Intervention - bicarbonate	Significance, *P*-value
**Number of patients**	584	304	280	
**HR, bpm, preoperative**	68 (67, 70)	68.5 (67, 70)	68 (67, 70)	0.875
**MAP, mmHg, preoperative**	96 (95, 98)	97 (95, 100)	95 (93, 97)	0.396
**MAP, mmHg, before induction**	85 (82, 85)	87 (85, 90)	80 (76, 83)	< 0.0001
**MAP, mmHg, 30 minutes before CPB**	70 (70, 71)	70 (70, 72)	70 (68, 71)	0.135
**MAP, mmHg, 30 minutes after CPB**	69 (68, 70)	70 (69, 71)	69 (67, 70)	0.109
**MAP, mmHg, end of surgery**	72 (71, 73)	73 (71, 74)	71 (70, 73)	0.150
**MAP, mmHg, ICU admission**	76 (75, 77)	76 (75, 78)	75 (73, 78)	0.339
**MAP, mmHg, 2 h after ICU admission**	73 (72, 74)	73 (71, 75)	73 (72, 74)	0.833
**MAP, mmHg, 4 h after ICU admission**	74 (72, 75)	74 (72, 76)	73 (72, 74)	0.541
**MAP, mmHg, 6 h after ICU admission**	73 (73, 74)	74 (73, 75)	73 (72, 75)	0.361
**MAP, mmHg, 8 h after ICU admission**	74 (73, 75)	74 (72, 75)	74 (72, 75)	0.632
**CVP, mmHg, after induction**	11 (10, 11)	11 (10, 11)	11 (10, 12)	0.340
**CVP, mmHg, 30 minutes before CPB**	8 (8, 8)	8 (8, 9)	8 (7, 9)	0.561
**CVP, mmHg, 30 minutes after CPB**	11 (10, 11)	10 (10, 11)	11 (10, 12)	0.663
**CVP, mmHg, end of surgery**	12 (11, 12)	12 (11, 12)	12 (11, 12)	0.396
**CVP, mmHg, ICU admission**	15 (15, 16)	16 (15, 16)	15 (15, 16)	0.689
**CVP, mmHg, 2 h after ICU admission**	15 (15, 16)	16 (15, 16)	15 (14, 15)	0.129
**CVP, mmHg, 4 h after ICU admission**	15 (15, 16)	15 (14, 16)	15 (15, 16)	0.425
**CVP, mmHg, 6 h after ICU admission**	14 (14, 15)	14 (14, 15)	14 (14, 15)	0.358
**CVP, mmHg, 8 h after ICU admission**	13 (13, 14)	13 (12, 14)	13 (12, 14)	0.801
**MPAP, mmHg, After induction**	25 (24, 27)	26 (24, 29) n = 61	25 (22, 29) n = 46	0.380
**MPAP, mmHg, 30 minutes after CPB**	25 (23, 27)	26 (23, 28) n = 76	25 (22, 27) n = 75	0.466
**MPAP, mmHg, 30 minutes after CPB**	26 (24, 27)	25 (23, 28) n = 104	26 (24, 28) n = 97	0.324
**MPAP, mmHg, end of surgery**	26 (25, 28)	26 (24, 28) n = 107	27 (24, 29) n = 94	0.659
**MPAP, mmHg, ICU admission**	29 (28, 30)	30 (29, 33) n = 113	27 (26, 29) n = 84	0.006
**MPAP, mmHg, 2 h after ICU admission**	30 (29, 31)	30 (29, 31) n = 120	29 (28, 31) n = 104	0.538
**MPAP, mmHg, 4 h after ICU admission**	30 (29, 31)	31 (29, 32) n = 126	29 (28, 30) n = 107	0.448
**MPAP, mmHg, 6 h after ICU admission**	29 (27, 29)	29 (27, 30) n = 125	28 (27, 30) n = 109	0.892
**MPAP, mmHg, 8 h after ICU admission**	28 (27, 29)	28 (26, 30) n = 128	28 (26, 30) n = 113	0.731
**CI, L/min/m2, after induction**	2.45 (2.24, 2.66)	2.46 (2.14, 2.77) n = 56	2.44 (2.17, 2.71) n = 38	0.947
**CI, L/min/m2, 30 minutes before CPB**	2.68 (2.57, 2.80)	2.62 (2.45, 2.79) n = 76	2.75 (2.58, 2.92) n = 75	0.269
**CI, L/min/m2, 30 minutes after CPB**	3.22 (3.08, 3.37)	3.14 (2.94, 3.34) n = 104	3.31 (3.09, 3.53) n = 97	0.261
**CI, L/min/m2, end of surgery**	3.02 (2.92-3.13)	2.95 (2.79, 3.11) n = 107	3.11 (2.97, 3.25) n = 94	0.140
**CI, L/min/m2, ICU admission**	2.80 (2.69, 2.92)	2.87 (2.73, 3.01) n = 113	2.71 (2.52, 2.89) n = 84	0.171
**CI, L/min/m2, 2 h after ICU admission**	3.06 (2.95, 3.17)	3.07 (2.92, 3.22) n = 120	3.05 (2.88, 3.21) n = 104	0.835
**CI, L/min/m2, 4 h after ICU admission**	3.12 (3.02, 3.23)	3.10 (2.98, 3.23) n = 126	3.15 (2.98, 3.32) n = 107	0.675
**CI, L/min/m2, 6 h after ICU admission**	3.24 (3.14, -3.35)	3.22 (3.10, 3.34) n = 125	3.27 (3.08, 3.46) n = 109	0.686
**CI, L/min/m2, 8 h after ICU admission**	3.18 (3.09, 3.28)	3.23 (3.11, 3.35) n = 128	3.13 (2.98, 3.28) n = 113	0.301
**SvO_2_, %, after induction (n = 118)**	79 (77, 80)	79 (78, 81) n = 61	77 (75, 79) n = 46	0.060
**SvO_2_, %, 30 minutes before CPB (n = 147)**	82 (81, 82)	82 (81, 83) n = 76	81 (80, 82) n = 75	0.357
**SvO_2_, %, 30 minutes after CPB (n = 177)**	78 (77, 79)	79 (77, 80) n = 104	78 (77, 80) n = 97	0.735
**SvO_2_, %, end of surgery (n = 179)**	77 (76, 78)	77 (76, 79) n = 107	77 (76, 79) n = 94	0.988
**SvO_2_, %, ICU admission (n = 192)**	71 (70, 72)	71 (70, 73) n = 113	70 (68, 72) n = 84	0.380
**SvO_2_, %, 2 h after ICU admission (n = 287)**	69 (68, 70)	69 (67, 70) n = 120	69 (67, 70) n = 104	0.931
**SvO_2_, % 4 h after ICU admission (n = 309)**	68 (67, 69)	67 (66, 69) n = 126	68 (67, 69) n = 107	0.322
**SvO_2_, %, 6 h after ICU admission (n = 316)**	69 (68, 70)	69 (68, 70) n = 125	69 (67, 70) n = 109	0.587
**SvO_2_, %, 8 h after ICU admission**	69 (68, 70)	69 (68, 71) n = 128	69 (67, 70) n = 113	0.264

Data are given as median/mean (95% confidence interval for the median/mean) as appropriate. HR: heart rate; MAP mean arterial blood pressure; CVP: central venous pressure; MPAP: mean pulmonary arterial pressure; CI: cardiac index; SvO_2_: mixed venous oxygen saturation. MPAP, CI, and SvO_2 _were not available in every patient.

**Table 5:** Renal function and metabolism

	Total cohort	Control - no bicarbonate	Intervention - bicarbonate	Significance, *P*-value
**Number**	**584**	**304 (52.1%)**	**280 (47.9%)**	
**Diuresis and fluid balance**				
Postoperative diuresis within 24 h, ml	2,844 (2,744, 2,944)	2,723 (2,564, 2,881)	2,977 (2,861, 3,092)	0.012
Postoperative cristalloid fluid balance, ml	-124 (-225, -22)	-133 (-295, 30)	-114 (-231, 4)	0.855
Postoperative surgical drainage, ml	927 (887, 968)]	933 (875, 991)	921 (865, 978)	0.782
Postoperative colloidals - drainage fluid balance, ml	549 (480, 618)	471 (374, 569)	633 (536, 731)	0.022
**Postoperative diuretics, number of patients**				
Furosemide	280 (47.9%)	142 (46.7%)	138 (49.3%)	0.590
Torasemide	462 (79.1%)	242 (79.6%)	220 (78.6%)	0.838
HCT	200 (34.2%)	102 (33.6%)	98 (35.0%)	0.779
Other diuretics	34 (5.8%)	20 (6.6%)	14 (5.0%)	0.524
**Renal function**				
Creatinine baseline, μmol/L	81 (79, 83)	81 (78, 84)	82 (78, 84)	0.787
Creatinine maximum*, μmol/L	93 (90, 96)	93 (88, 97)	93 (89, 97)	0.785
Creatinine at discharge*, μmol/L	82 (80, 87)	84 (80, 88)	80 (80, 87)	0.153
Maximum relative increase in creatinine*				
< 25%	369 (63.2%)	204 (67.1%)	165 (58.9%)	0.217
> 25%	123 (21.1%)	57 (18.8%)	66 (23.6%)	
> 50%	56 (9.6%)	25 (8.2%)	31 (11.1%)	
> 100%	36 (6.2%)	18 (5.9%)	18 (6.4%)	
eGFR baseline, ml/min	82 (80, 84)	81 (78, 84)	83 (80, 87)	0.434
eGFR minimum*, ml/min	69 (67, 71)	69 (66, 72)	70 (66, 73)	0.744
eGFR at discharge*, ml/min (n = 566)	82 (79, 85)	81 (77, 86)	83 (79, 86)	0.586
AKI grading according to creatinine changes (new RRT = grade 3), number patients				
Grade 1	85 (14.6%)	38 (12.5%)	47 (16.8%)	0.478
Grade 2	7 (1.2%)	3 (1.0%)	4 (1.4%)	
Grade 3	74 (12.7%)	39 (12.8%)	35 (12.5%)	
AKI grading according to diuresis** (new RRT = grade 3), number patients				
Grade 1	14 (2.4%)	11 (3.6%)	3 (1.1%)	0.135
Grade 2	1 (0.2%)	0 (0.0%)	1 (0.4%)	
Grade 3	45 (7.7%)	21 (6.9%)	24 (8.6%)	
AKI grading, total**, number patients				
Grade 1	90 (15.4%)	44 (14.5%)	46 (16.4%)	0.753
Grade 2	8 (1.4%)	3 (1.0%)	5 (1.8%)	
Grade 3	75 (12.8%)	39 (12.8%)	36 (12.9%)	
New RRT				
Patients (%)	45 (7.7%)	22 (7.2%)	23 (8.2%)	0.774
Duration of treatment, h	144 (114, 196)	153 (110, 196)	125 (76, 227)	0.892
**Intraoperative metabolism**				
Blood glucose max, mg/dl	193 (189, 202)	189 (184, 193)	197 (193, 204)	0.005
Blood glucose min, mg/dl	92 (91, 93)	91.5 (89, 94)	92 (91, 94)	0.181
pH max	7.45 (7.44, 7.45)	7.45 (7.45, 7.45)	7.45 (7.44, 7.45)	0.034
pH min	7.32 (7.32, 7.32)	7.32 (7.31, 7.32)	7.32 (7.32, 7.33)	0.157
Lactate max, mmol/L	1.7 (1.6, 1.8)	1.6 (1.5, 1.7)	1.8 (1.6, 1.9)	0.005
Lactate min, mmol/L	0.6 (0.6, 0.6)	0.6 (0.6, 0.7)	0.7 (0.6, 0.7)	0.594
**Postoperative metabolism**				
Blood glucose max, mg/dl	169.0 (166.0, 172.0)	163.5 (161.0, 167.0)	173.5 (170.12, 177.88)	0.0015
Blood glucose min, mg/dl	90.09 (88.68,1.51)	89.70 (87.73, 91.67)	90.53 (88.48, 92.57)	0.566
pH max	7.46 (7.46, 7.47]	7.46 (7.45, 7.46)	7.47 (7.46, 7.48)	0.001
pH min	7.32 (7.31, 7.32)	7.32 (7.31, 7.32)	7.32 (7.31, 7.33)	0.165
Lactate max, mmol/L	2.00 (1.90, 2.10)	1.90 (1.70, 2.00)	2.10 (2.00, 2.20)	0.010

Fluid balance, diuretics, metabolism, and renal function are shown for patients undergoing elective on-pump cardiac surgery with or without a perioperative infusion of bicarbonate. HCT: hydrochlorothiazide; eGFR: estimated glomerular filtration rate: modifications of diet in renal disease formula; AKI: acute kidney injury; CVVH: continuous-veno-venous hemofiltration; max: maximal; min: minimal. Data are given as absolute numbers (percentage) for nominal variables, median (95% confidence interval) for non-normally distributed data, and mean (95% confidence interval) for normally distributed variables. *Changes in plasma creatinine levels and derived variables were calculated after exclusion of patients treated with renal replacement therapy. **AKI grading was performed according to Acute Kidney Injury Network recommendations (9) on the basis of changes in plasma creatinine, need for new renal replacement therapy and time course of diuresis (within 48 h after surgery).

### Intra- and postoperative treatments

While no significant differences in the need for mechanical circulatory support were observed (Table 2), hemodynamic treatments differed markedly between the groups. Patients in the BIC group were treated with more crystalline and colloidal fluids, and more frequently and with higher doses of noradrenaline (Table 3). No differences were observed in the use of inotropic drugs. No differences in the rate of transfusion of packed red cells, fresh frozen plasma, or thrombocyte concentrates were observed.

### Renal outcomes

Patients in the BIC group had a higher diuresis within the first 24 h after surgery. No differences were observed in the use of diuretics (Table 5). Renal replacement therapy (RRT) was initiated in 7.2% of patients in the CON and 8.2% of patients in the BIC group. Time to initiation of RRT after ICU admission was 29.0 (19.0 to 39.0) h in the CON and 35.5 (24.5 to 49.0) h in the BIC group (*P *not significant). The specific changes in plasma creatinine, eGFR and the grading according to the Acute Kidney Injury Network criteria are depicted in Table 5.

### General clinical outcomes

Despite comparable postoperative ventilation times, duration of treatment in the ICU and in the high dependency unit (HDU) were significantly prolonged in the BIC group (Table 6). No differences in direct or indirect measures of morbidity and mortality were observed (Table 6).

**Table 6:** Clinical course and complications

	Total cohort	Control - no bicarbonate	Intervention - bicarbonate	Significance, *P*-value
**Number**	584	304 (52.1%)	280 (47.9%)	
**Postoperative ventilation time, h**	7 (7, 7)	7 (6, 8)	7 (7, 8)	0.234
**Reintubation**	46 (7.9%)	22 (7.2%)	24 (8.6%)	0.648
**Tracheostomy**	18 (3.1%)	8 (2.6%)	11 (3.6%)	0.677
**Duration in ICU, h**	23 (23, 24)	23 (22, 24)	24 (23, 24)	0.093
**Duration in IMC, h**	38 (27, 43)	27 (24, 42)	44 (36, 48)	0.0008
**Duration in HDU, h**	85 (73, 89)	83 (71, 88)	88 (72, 96)	0.026
**Re-admission to ICU**	19 (3.3%)	9 (3.0%)	10 (3.6%)	0.849
**Need for CPR**	10 (1.7%)	6 (2.0%)	4 (1.4%)	0.851
**Reoperation**	80 (13.7%)	45 (14.9%)	35 (12.5%)	0.492
**MAC Score**				
1	54 (9.2%)	25 (8.2%)	29 (10.4%)	0.728
2	25 (4.3%)	14 (4.6%)	11 (3.9%)	
3	10 (1.7%)	5 (1.6%)	5 (1.8%)	
4	1 (0.2%)	0 (0.0%)	1 (0.4%)	
**Status upon discharge from hospital**				
Extubated	551 (94.3%)	290 (95.4%)	261 (93.2%)	0.377
Intubated	10 (1.7%)	2 (0.7%)	8 (2.9%)	
Tracheostomy	13 (2.2%)	7 (2.3%)	6 (2.1%)	
Died in hospital	8 (1.4%)	4 (1.3%)	4 (1.4%)	
**Status after 30 days**				
Dead	11 (1.9%)	5 (1.6%)	6 (2.1%)	0.573
Alive	572 (97.9%)	298 (98.0%)	274 (97.9%)	
Unknown	1 (0.2%)	1 (0.3%)	0 (0.0%)	

Postoperative course, complications, and clinical outcomes are shown in patients undergoing elective on-pump cardiac surgery with or without a perioperative infusion of bicarbonate. CPR: cardiopulmonary resuscitation; MAC score: 1 point for each of the following complications - low cardiac output syndrome, stroke, new need for renal replacement therapy, reintubation; IMC: intermediate care unit; HDU: high dependency unit. Data are given as absolute numbers (percentage) for nominal variables, median (95% confidence interval) for non-normally distributed data, and mean (95% confidence interval ) for normally distributed variables.

## Discussion

AKI is not only a frequent complication in cardiac surgical patients [1] but has also been shown to be independently associated with morbidity and mortality [10,11]. Unfortunately, little progress has been made within the last years in the development of strategies to reduce the incidence and improve the prognosis of this complication.

Recently, Haase and coworkers have elegantly delineated a pathophysiological line of evidence that the severity of the renal insult induced by on-pump cardiac surgery may, at least in part, be related to the degree of hemoglobinuria: the histological features of CSA-AKI resemble the pigment nephropathy typically observed during rhabdomyolysis [5]. Since alkalization of the urine is among the established measures to treat rhabdomyolysis [12] they used this concept successfully as a strategy for the prevention of CSA-AKI in a small pilot trial [7]. With respect to these promising findings, the relatively high incidence of CSA-AKI at our institution, the lack of other available measures for preventing renal dysfunction during cardiac surgery [13], and the fact that urine alkalization for the treatment of rhabdomyolysis has a longstanding tradition in clinical medicine [12], we chose to implement this concept into our clinical routine. It is of note that an interdisciplinary working group on this topic also gave a positive recommendation to use hydration and bicarbonate to reduce the nephrotoxic effects of myo- and hemoglobinuria [14].

In contrast to these promising findings, the results of the present prospective observational cohort study show that, in a heterogeneous patient population and under the real life conditions of a University hospital, perioperative treatment with BIC does not reduce the incidence of CSA-AKI as measured by postoperative changes in creatinine, and the need for dialysis. Moreover, it is associated with clearly unwarranted effects like a decrease in arterial blood pressure (during the bolus application of BIC), an increased need for fluids and vasopressors, and an increased need for treatment in the HDU.

The BIC dose chosen at our institution was comparable to the dose used by Haase and coworkers in their pilot study [7]. However, they did not observe any adverse effects or differences in the perioperative use of fluids. Unfortunately, the doses of vasopressors and inotropes used are not presented in this study.

With respect to the observational nature of our study, we cannot completely rule out the possibility that the adverse effects observed during the intervention period were related to other, unmeasured variables. However, the patient groups were ideally matched at baseline; not only with respect to conventional demographic factors and surgical procedures but also regarding the plasma levels of established cardiovascular risk markers. The patients were closely followed during the perioperative course, and a relevant number of patients were monitored invasively with a pulmonary arterial catheter to determine the cardiac index and SvO2. We are also not aware of any other clinical factor that was changed during this time: neither the introduction of another, new clinical treatment nor major changes in the treatment team. Thus it is rather likely that the observed adverse effects in the intervention period were indeed related to the use of bicarbonate infusion, that is, alkalization, despite only achieving minimal changes in maximal plasma pH.

Data show that BIC is frequently used in cardiac surgical patients to treat acidosis, especially during cardiopulmonary bypass. Despite this, few data are available on the short- and long-term hemodynamic effects of BIC. Tripathi and coworkers observed a biphasic response after the infusion of 1 mmol BIC/Kg BW during steady state conditions during CPB with an immediate venous pooling (leading to a decrease in the CPB reservoir volume), followed by a moderate increase in MAP. In contrast, observations in patients with end-stage renal disease show that higher dialysate bicarbonate concentrations lead to a decrease in arterial blood pressure during dialysis [15], an effect that may be explained by an increase in endothelial nitric oxide production [16].

Since the differences in maximal pH between the study groups were rather small (0.1 pH difference) despite being statistically significant, one may assume that the lack of a nephroprotective effect of bicarbonate infusion may be related to the fact that no adequate alkalization was achieved. It is of note that Haase and coworkers observed a mean difference in pH of 0.7 (7.38 to 7.45) [7] between the intervention and the control group with the same dose of bicarbonate. This discrepancy may be related to the fact that we did not record mean pH in the intra- and postoperative period, which would probably have better reflected the differences in this variable in comparison between the control and the intervention group.

As an important difference in comparison with the previous pilot study [7] we did not specifically treat only patients at risk for CSA-AKI, since the identification of such patients is not trivial with respect to the multifactorial nature of this complication. Median CPB duration, an accepted risk factor for CSA-AKI, was relatively long in the present study population, suggesting that our patients may be regarded as at risk per se. Nonetheless, the risk for AKI attributable to prolonged CPB is probably lower than the risk of selected groups of patients presenting with multiple risk factors for AKI. Consequently our findings do not rule out the possibility that a perioperative BIC infusion may be nephroprotective in selected, especially vulnerable cardiac surgery patients, as are currently being recruited for the BIC-NC study.

Comparably, we cannot rule out that the minor effect of the BIC infusion on plasma pH in our study in comparison with Haase's work may be related to the different types of crystalloid infusions used: 0.9% sodium chloride in the Australian setting [7] and a balanced electrolyte infusion containing 24 mmol/L actetate in our study. Thus, one may speculate that the use of balanced fluids may reduce the effectiveness of BIC as a measure to prevent CSA-AKI, in comparison with the clinical setting in Australia in which patients were treated with more acidic solutions [7]. Interestingly, acetate itself does also have vasodilating properties [17]. Consequently, the higher doses of crystalloid solutions used in the intervention group may also have aggravated the vasodilatation (that is, the higher need for vasopressors) observed in the intervention period.

### Limitations

Despite all efforts to follow the patients meticulously and to rule out other factors that may have influenced our results, the design of this study as an observational cohort study has a substantial risk of bias. The present ongoing BIC-NC study (Clinical trials identifier NCT00672334) will help to elucidate further, the risks and benefits of using BIC to prevent AKI in patients undergoing cardiac surgery.

With respect to the observational nature of this study we did not perform an a priori power analysis to determine if the sample size is adequate. However, we enrolled more patients than planned for the BIC-NC study and additionally tested the endpoint, namely, 25% increase in plasma creatinine, suggesting that our dataset is adequately powered.

## Conclusion

In conclusion, the present prospective observational trial failed to reproduce the beneficial effects of a 24-h perioperative infusion of 4 mmol/Kg BIC on the incidence of AKI in patients undergoing cardiac surgery, that were recently shown in a small pilot trial. Routine treatment in a heterogeneous cohort with BIC was associated with an increased need for fluids and vasopressors. If this is a direct effect of BIC infusion, or the indirect effect of an associated treatment with balanced crystalloid solutions containing the vasodilating buffer acetate, merits further investigation.

## Key messages

• The routine perioperative infusion of 4 mmol /Kg BW sodium failed to reduce the incidence of acute kidney injury and the need for renal replacement therapy in a heterogeneous cohort of cardiac surgical patients.

• Treatment with BIC was associated with hypotension, a higher need for vasopressors, and a prolonged stay in the HDU but had no other significant adverse effects on non-renal morbidity and 30-day mortality.

## Abbreviations

AKI: acute kidney injury; BIC: sodium bicarbonate; CABG: coronary artery bypass grafting; CI: cardiac index; CON: control (non-intervention) group; CPB: cardiopulmonary bypass; CSA-AKI: cardiac surgery-assocaiated acute kidney injury; CVP: central venous pressure; eGFR: estimated glomerular filtration rate; HDU: high dependency unit; HR: heart rate; hsTNT: high-sensitivity troponin T; ICU: intensive care unit; IMC: intermediate care unit; MAP: mean arterial pressure; NTproBNP: N-terminal pro B-type natriuretic peptide; PAP: pulmonary arterial pressure; RRT: renal replacement therapy; ScO2: cerebral oxygen saturation; ScvO2: central venous oxygen saturation; SvO2: mixed venous oxygen saturation.

## Competing interests

The authors declare that they have no competing interests.

## Authors' contributions

MH, HH, JH and TH designed the study, supervised the statistical analyses and drafted the manuscript. MS, YN, JG, MK, and HP collected the data and performed the statistical analyses. All authors read and approved the final manuscript.
